# Successful treatment of 2 imported cases of *Mansonella perstans* infection

**DOI:** 10.1371/journal.pntd.0005452

**Published:** 2017-05-25

**Authors:** Hilmir Asgeirsson, Andreas Harling, Silvia Botero-Kleiven

**Affiliations:** 1 Department of Infectious Diseases, Karolinska University Hospital, Stockholm, Sweden; 2 Unit of Infectious Diseases, Department of Medicine Huddinge, Karolinska Institutet, Stockholm, Sweden; 3 Department of Infectious Diseases, Karlstad Central Hospital, Karlstad, Sweden; 4 Department of Clinical Microbiology, Karolinska University Hospital, Stockholm, Sweden; George Washington University, UNITED STATES

## Introduction

*Mansonella perstans* is a human filarial parasite endemic in many countries of sub-Saharan Africa, as well as in parts of South and Central America. *M*. *perstans* is spread by biting midges of the genus *Culicoides*. The adult parasites are thought to live in serous body cavities, and the female parasites release microfilariae into the blood [1, 2]. The clinical manifestations of *M*. *perstans* infection are poorly defined, but possible symptoms are swellings in extremities or face, itching, rash, exhaustion, and pain from serous cavities [1–4]. Few recent reports exist that describe the symptoms or treatment of travelers and migrants with *M*. *perstans* infection [5–8]. With increasing migration and travel, an increase in masonelliasis cases diagnosed in nonendemic areas may, however, be expected. *M*. *perstans* has been regarded as one of the most difficult filarial infections to treat. Antihelminthig drugs that have been tried against the infection include diethylcarbamazine (DEC), the benzimidazoles (e.g., albendazole and mebendazole), and ivermectin [1, 9, 10]. After the discovery of the symbiotic bacteria *Wolbachia* in *M*. *perstans* strains from certain endemic regions, doxycycline has, however, emerged as a promising treatment alternative [11, 12]. Here, the symptoms and treatment of 2 imported cases of *M*. *perstans* infection are described.

## Presentation of cases

### Case 1

**Fig 1:**
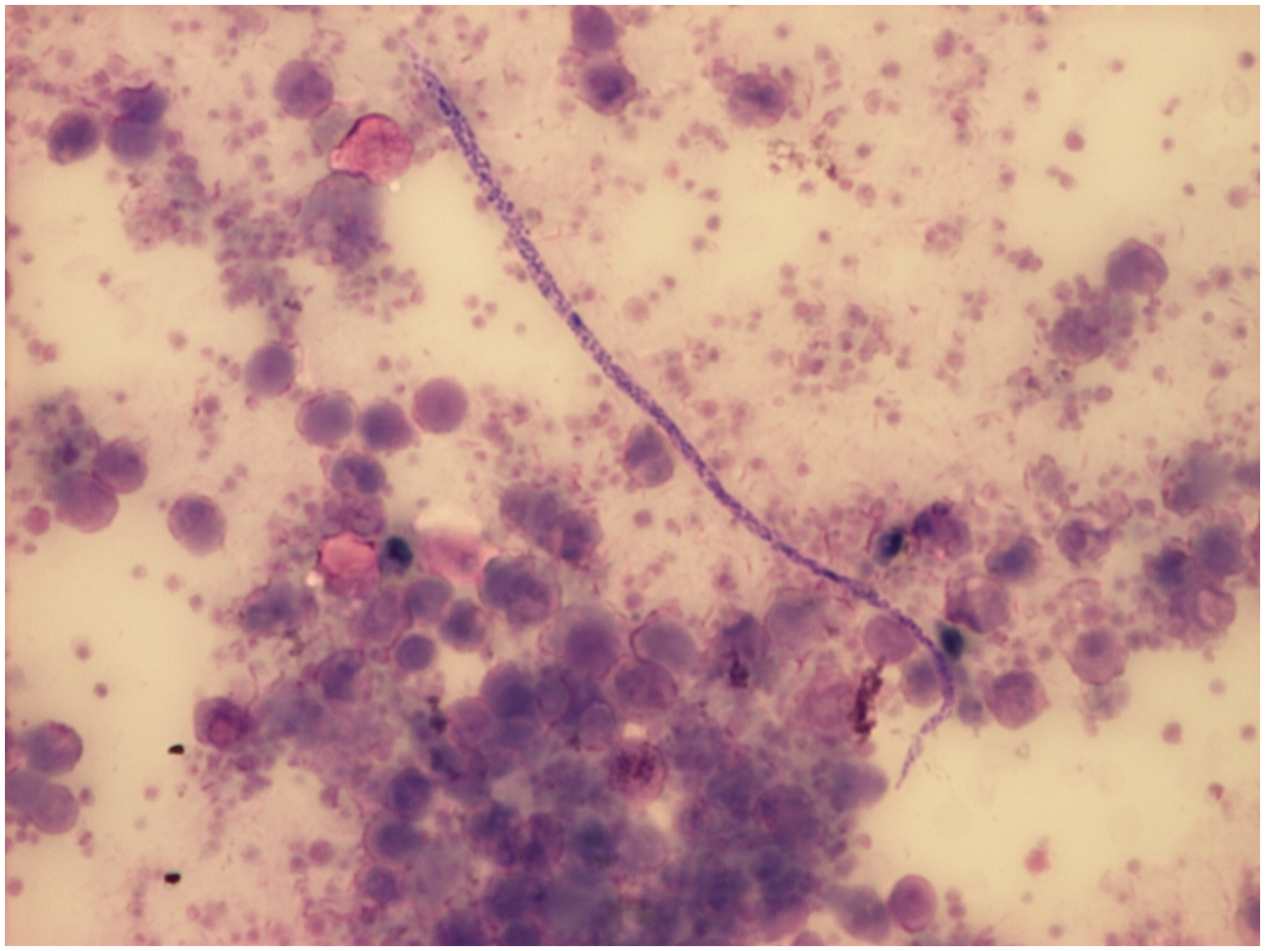
*Mansonella perstans* microfilaria as seen in microscopy of peripheral blood sediment after Knott’s concentration (Giemsa stain, 200X). Microfilariae were unsheathed and measured approximately 200 x 4 μm, the nuclei extending to the tip of the characteristically blunt tail. (Source: Silvia Botero).

A 57-year-old woman complained of 4–6 weeks of fatigue, feeling feverish, and extensive night sweats and 3 weeks of coughing and dyspnea. She had returned from Mozambique 2 weeks earlier, where she had been living for the past 5 years. She was of Swedish origin, previously healthy, took no medications, and had no known allergies. The white blood cell count was 37.3 x 10^9^/L, with eosinophils 24.8 x 10^9^/L. Serum immunoglobulin E (IgE) was 220 kE/L. Antineutrophil cytoplasmic antibodies were not present. Computed tomography of the thorax and abdomen visualized bilateral peribronchial infiltrates. Bone marrow biopsy revealed a cell-rich marrow with high-grade eosinophilia, without suspicion of malignancy. Multiple stool and urine analyses for ova and parasites, including cultures for *Strongyloides*, did not identify any pathogens. Peripheral blood microscopy (membrane filtration technique) revealed *M*. *perstans* 1 microfilaria/ml (Fig 1). Filaria serology (in-house ELISA based on cross-reaction of antibodies directed against common *Acanthocheilonema vitae* antigens) was strongly positive (209 arbitrary units, cutoff 17), while for *Strongyloides*, optical density (OD) of 1.5 units (cutoff 0.5) was a suspected cross-reaction. Serological analyses for *Fasciola*, *Schistosoma*, *Toxocara*, and *Trichinella* were all negative.

Prednisolone 30 mg/day was initiated and tapered over 7 weeks. Concurrently, doxycycline 200 mg/day was prescribed for 6 weeks. Initially, the symptoms improved and the eosinophil count normalized, but shortly after having finished the doxycycline and prednisolone treatments, the patient again complained of increasing fatigue. Blood test revealed that the eosinophils had risen to 7.9 x 10^9^/L, and repeat blood microscopy showed *M*. *perstans* 3 microfilariae/ml. Prednisolone again had good symptomatic effects (tapered over 12 weeks). Albendazole 1,000 mg/day (13 mg/kg/day in 2 doses) was prescribed for 4 weeks, combined with ivermectin 15 mg and a second dose 4 months later. Microscopy for microfilariae 8 months after the second ivermectin dose was negative. Nine months later (2 years after the first contact), the patient was still feeling well and had a negative microscopy. The filaria serology titer had decreased to 42 units, with normalized serum IgE (63 kE/L) and eosinophil count (0.4 x 10^9^/L).

### Case 2

A previously healthy 40-year-old male presented with fever, fatigue, loose stools, and mild pruritus 1 month after immigrating from the Democratic Republic of Congo (DRC). *Plasmodium vivax* malaria was diagnosed and chloroquine phosphate prescribed. As an incidental finding during malaria microscopy, microfilariae were noticed. The eosinophil count was 0.5x10^9^/L. Most of the symptoms improved after malaria treatment, but he continued complaining of fatigue and itching. Further analysis revealed *M*. *perstans* 83 microfilariae/ml and *Loa loa* 670 microfilariae/ml peripheral blood (membrane filtration technique). Filaria ELISA was positive, 1.19 OD (cutoff 0.5). Stool and urine analyses for ova and parasites were negative. Doxycycline 200 mg/day for 6 weeks and albendazole 400 mg/day (6 mg/kg/day) for 3 weeks were initiated, combined with ivermectin 12 mg after 4 weeks and a second dose after 3 months (considered safe to use, since *L*. *loa* microfilaremia level was not high)[13]. Blood microscopy 10 weeks after the doxycycline treatment was still positive for *L*. *loa* (130 microfilariae/ml) but negative for *M*. *perstans*. A 3-week albendazole treatment was repeated, with 2 doses ivermectin (diethylcarbamazine, usually regarded as the drug of choice for loiasis, is not easily available in Sweden). Microscopy 3 months later (10 months after the first contact) showed decreased *L*. *loa* microfilaremia (<1 microfilariae/ml) and remained negative for *M*. *perstans*. Sixteen months after the first contact, the patient was still feeling well and had negative microscopy for microfilariae.

## Discussion

Here, we have described 2 cases of *M*. *perstans* infection imported to Europe and successfully treated with combination treatments including doxycycline, albendazole, and ivermectin. In both cases, microfilariae were identified by blood microscopy, and filaria serology was positive. In the first patient, the amount of microfilariae in blood was very low, but it nevertheless caused a severe hypersensitivity reaction. Although uncommon, high-grade eosinophilia has previously been reported in association with *M*. *perstans* infection in travelers [5–7]. In the second patient, the only symptoms were mild itching and fatigue. Hence, both patients had unspecific symptoms with various possible differential diagnoses. This highlights the importance of awareness of *M*. *perstans* infection in travelers and migrants coming from endemic areas.

It is not known how commonly *M*. *perstans* strains in Mozambique and DRC harbor *Wolbachia*. If our patients’ strains harbored *Wolbachia*, doxycycline treatment alone would possibly have been sufficient. Doxycycline is thought to have an effect on adult parasites and on female parasite fertility and embryogenesis rather than having direct effect on microfilariae [11]. Therefore, disappearance of *M*. *perstans* microfilariae from the blood should not be expected directly following doxycycline therapy (because of the life span of the microfilariae). However, albendazole (possible effect on adult parasites) and ivermectin (possible effect on microfilariae) were added in the first case because of the severity of disease and persisting hypereosinophilia and in the second case because of the concomitant *L*. *loa* infection. The cases nevertheless illustrate that it is possible to clear *M*. *perstans* microfilaremia with antihelminthic therapy containing doxycycline.

In conclusion, hypersensitivity reaction, possibly severe, with eosinophilia may be a manifestation of *M*. *perstans* infection in travelers and migrants coming from endemic areas. Treatment regimens including doxycycline may be effective in clearing *M*. *perstans* microfilariae.

**Key learning points**

- *M*. *perstans* infection commonly presents with unspecific symptoms.
- *M*. *perstans* infection may present as hypersensitivity reaction, possibly severe, with eosinophilia in travelers and migrants coming from endemic areas.
- Antihelminthic treatment regimens including doxycycline may be effective in clearing *M*. *perstans* microfilariae.
